# Ultrastructural Proof of Polyomavirus in Merkel Cell Carcinoma Tumour Cells and Its Absence in Small Cell Carcinoma of the Lung

**DOI:** 10.1371/journal.pone.0004958

**Published:** 2009-03-23

**Authors:** Charlotte T. A. H. Wetzels, Jolanda G. M. Hoefnagel, Judith M. J. E. Bakkers, Henry B. P. M. Dijkman, Willeke A. M. Blokx, Willem J. G. Melchers

**Affiliations:** 1 Department of Pathology, Radboud University Nijmegen Medical Centre, Nijmegen, the Netherlands; 2 Department of Medical Microbiology, Radboud University Nijmegen Medical Centre, Nijmegen, the Netherlands; The University of Queensland, Australia

## Abstract

**Background:**

A new virus called the Merkel Cell Polyomavirus (MCPyV) has recently been found in Merkel Cell Carcinoma (MCC). MCC is a rare aggressive small cell neuroendocrine carcinoma primarily derived from the skin, morphologically indistinguishable from small cell lung carcinoma (SCLC). So far the actual presence of the virus in MCC tumour cells on a morphological level has not been demonstrated, and the presence of MCPyV in other small cell neuroendocrine carcinomas has not been studied yet.

**Methodology/Principal Findings:**

We investigated MCC tissue samples from five patients and SCLCs from ten patients for the presence of MCPyV-DNA by PCR and sequencing. Electron microscopy was used to search ultrastructurally for morphological presence of the virus in MCPyV-DNA positive samples. MCPyV was detected in two out of five primary MCCs. In one MCC patient MCPyV-DNA was detected in the primary tumour as well as in the metastasis, strongly suggesting integration of MCPyV in the cellular DNA of the tumour in this patient. In the primary MCC of another patient viral particles in tumour cell nuclei and cytoplasm were identified by electron microscopy, indicating active viral replication in the tumour cells. In none of the SCLCs MCPyV-DNA was detected.

**Conclusions/Significance:**

Our results strongly suggest that MCPyV is an oncogenic polyomavirus in humans, and is potentially causally related to the development of MCC but not to the morphological similar SCLC.

## Introduction

Merkel Cell Carcinoma (MCC) is a rare carcinoma of the skin that metastasizes quickly and has a 5-year mortality rate as high as 50% [1]. The incidence is dramatically increasing and has tripled in the past 15 years in the U.S. [2]. Factors implicated in the etiology are ultraviolet exposure, advanced age and immunosuppression [2]. In particular, many forms of T-lymphocyte immune suppression are linked to MCC, e.g. AIDS [3], solid organ transplantation [4] and chronic lymphocytic leukaemia [5].

In 2008 Feng et al. discovered a new polyomavirus in MCC tissue samples [6], and called it the Merkel cell polyomavirus (MCPyV). The family of Polyoma viruses is subdivided into several genetically distinct groups. One of these groups, the SV40 subgroup, contains the four known human Polyoma viruses: BK, JC, KI and WU. BK and JC virus are known to cause infections in immunocompromised patients, while KI and WU were isolated from nasopharyngeal secretions of patients with respiratory infections [7]–9. Polyomaviruses can be oncogenic in animals, but until now, no polyomavirus has been proven to be oncogenic in humans [10].

Feng et al. showed that the MCPyV is integrated at different locations within the human genome in the MCC, and furthermore that the large T antigen transcript is expressed in MCC [6]. This implicates that MCPyV is not only associated with MCC, but that it might in fact be the causative agent. Indeed, several studies have reported the presence of MCPyV-DNA in 43–85% of MCC cases [6], [11]–[13].

MCC and small cell lung carcinoma (SCLC) are both aggressive neuroendocrine carcinomas, histologically composed of morphologically identical small cells. In fact, MCC is often referred to as the cutaneous form of small cell lung carcinoma [14]. Like MCC, SCLC also is a highly malignant carcinoma with a 5-year survival rate lower than 5% [15]. This tumour comprises about 10–20% of the lung cancers and is strongly related to tobacco usage [15], [16].

To investigate the incidence of MCPyV and the relation to oncogenesis, tissue samples from small cell neuroendocrine carcinomas from both skin and lung were analyzed for the presence of MCPyV-DNA. We found that MCPyV-DNA was present in 40% of the MCC cases, and was absent in all SCLCs. This suggests that MCPyV has a restricted tropism. MCPyV-DNA was found in the primary and the metastatic tumour of one MCC patient, indicating viral DNA integration. Furthermore, we studied MCC tissue samples by electron microscopy in order to determine the actual presence of viral particles in the tumour cells. MCPyV viral particles were ultrastructurally identified in primary MCC tumour cell cytoplasm and nuclei in one patient, strongly suggesting active viral replication.

## Materials and Methods

### Patients and tissues

We obtained frozen and paraffin-embedded (formalin fixed) MCC and SCLC biopsy and resection samples from the archives of the Pathology department of the Radboud University Nijmegen Medical Centre. All samples were collected for diagnostic purposes in the years 1995–2008. From five MCC patients seven samples were used, including two primary MCCs and their metastases. Frozen SCLC samples from ten patients were used, representing three primary tumours, three nodal and four distant metastases. In all cases the diagnosis of either MCC or SCLC was confirmed with immunohistochemistry using thyroid transcription factor-I and low-molecular weight cytokeratin 20 in order to distinguish between the two entities. For PCR, control specimens consisting of frozen and paraffin-embedded tissue samples from eight patients with variable malignant and benign skin diseases were used. The clinical characteristics of all patients are listed in Table 1.

**Table 1:** Patient Characteristics.

Patient ID	Gender	Age	Location	Immunosuppressed	Diameter (mm)	Solar elastosis
*Merkel Cell Carcinoma*						
1	f	72	Facial skin	No	6	Yes
2	m	56	Tongue	No	12	No
3a	m	74	Facial skin	No	15	Yes
3b			Metastasis skin lower leg		unknown	
4	f	65	Facial skin	No	13	Yes
5a	m	47	Facial skin	Renal transplant	>50	Yes
5b			Liver metastasis			

*Small Cell Lung Carcinoma*				
1	f	55	Mediastinal metastasis	No
2	f	72	Intrapulmonary	No
3	m	59	Cerebral metastasis	No
4	f	40	Mediastinal metastasis	No
5	m	62	Lymph node metastasis	No
6	m	58	Cerebral metastasis	No
7	f	67	Hepatic metastasis	No
8	f	48	Intrapulmonary	No
9	m	63	Intrapulmonary	No
10	m	64	Cerebral metastasis	No

*Control patients (skin lesions)*					Type of lesion
1	m	62	Face	No	Basal cell carcinoma
2	m	55	Face	No	Verruca seborrhoica
3	f	37	Mamma	No	Dermal naevus
4	m	54	Digit	Renal transplant	Squamous cell carcinoma
5	f	69	Leg	No	Psoriasiform dermatitis
6	m	63	Hand	No	Spongiotic dermatitis
7	f	66	Back	No	Porphyria
8	f	45	Presternal	No	Dermal naevus

f: female; m: male.

### DNA isolation

The paraffin embedded sections were incubated overnight with Proteinase K in lysis buffer (56°C) before DNA was isolated using the EZ1 DNA Tissue Kit (Qiagen Benelux BV) as described previously [17]. The DNA was isolated from the frozen tissue sections by using Magnapure Total NA isolation kit (Roche Molecular Diagnostics). The isolation product was stored at −80°C until used for PCR.

### PCR and sequence analysis

For PCR, 4 µm sections were used of the paraffin embedded tissue, and 10 µm sections of the frozen tissue samples. All samples were prepared and analyzed under sterile conditions. All tissue samples were studied in accordance with national ethical principles.

Before MCPyV-DNA detection, the quality of the DNA was tested by β-globin PCR for human DNA detection (Roche Diagnostics). All samples tested positive. The primers for MCPyV detection were adapted from Feng et al. [6]. We used LT3 as this primer set was reported to have the highest sensitivity [13]. We decreased the size of the amplicons to 250 bp to obtain higher sensitivities in paraffine processed tissue samples [18]. The forward primer sequence was 5′-atc tgc acc ttt tct aga ctc c-3′, and the reverse primer sequence 5′-ata tag ggg cct cgt caa cc-3′. The MCPyV PCR was performed using PCR Mastermix (Roche Diagnostics), containing Taq DNA Polymerase. Five µL of DNA was used in a total volume of 45 µL. Water instead of DNA template was used for PCR-negative controls containing all other PCR components. The PCR consisted of 40 cycles with an annealing temperature of 50°C. The PCR products were analyzed by gel electrophoresis, and by sequence analysis.

The MCPyV positive samples were sequenced using the Big Dye Terminator v3.1 Cycle Sequence (Applied Biosystems). The obtained sequences were compared with the reference sequence of the National Center for Biotechnology Information (NCBI) Entrez Nucleotide database (gb/EU375803.1 Merkel cell polyomavirus isolate MCC350 and gb/EU375804.1 Merkel cell polyomavirus isolate MCC339), using the NCBI blast program. Confirmed positive samples were later used as positive controls in subsequent PCR experiments.

### Electron microscopy

For electron microscopy a piece of paraffin embedded material from two MCPyV positive primary MCC samples was used. The material was deparaffinised overnight in xylene, rehydrated and washed in 0.1 M sodium cacodylate buffer. The tissue fragments were postfixed in palade-buffered 2% OsO4 for 1 h, dehydrated, and embedded in Epon812, Luft's procedure (Merck, Darmstadt, Germany). Ultrathin sections were contrasted with 4% uranyl acetate for 45 min and subsequently with lead citrate for 5 min at room temperature. Sections were examined in a Jeol 1200 EX2 electron microscope (JEOL, Tokyo, Japan).

#### Ethics statement

The tissue samples were originally taken for diagnostic purposes. For our research we selected the remainder of tissue samples from our archive after which they were encoded. Our hospital uses the policy that encoded patient material that was originally removed for diagnostic purposes may be used for further research unless the patient has stated otherwise. In case of research with encoded/anonymous material informed consent is not required, as long as the researcher is not able to discover the patients identity linked to the research material. When a patient has explicitly refused, it is not allowed to use these tissue samples for research. The authors state that they used encoded tumor samples, and that none of the patients explicitly refused to participate in research.

The researchers did not consult an ethics committee/IRB prior too the research.

## Results

The mean age of MCC patients was 63 years and 60% were male. MCPyV-DNA was detected in the primary tumours of two out of five MCC patients (40%). Of these two patients one had metastatic cancer, and MCPyV-DNA was detected in this metastasis as well (patient no. 3). The other three primary MCC samples and the metastasis of the other metastasised patient, were all tested negative for MCPyV-DNA. Sequencing of all positive PCR products showed MCPyV-DNA.

**Figure 1:**
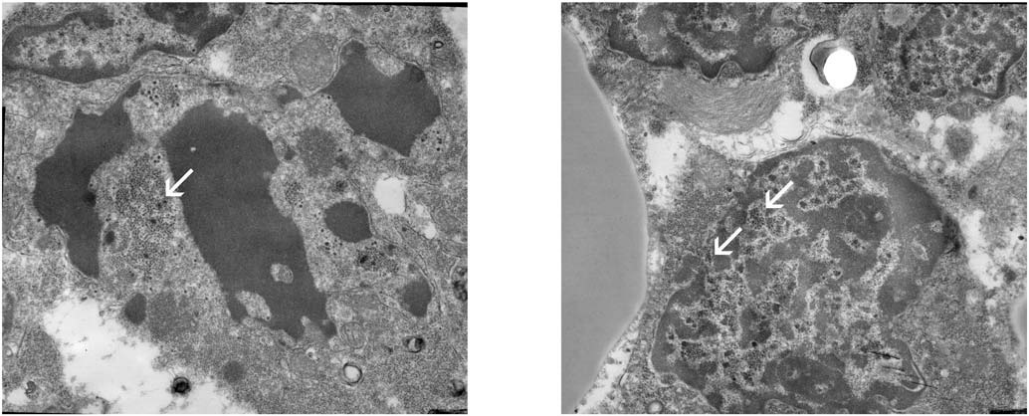
Electron micrographs showing viral particles in a sample of patient no. 4. a) destroyed merkel cell carcinoma cell with viral particles (white arrow) measuring 50 nm, mingled with nuclear fragments and multiple smaller ribosomes (20 nm). b) penetration of virions through the nuclear membrane (white arrows) toward the cytoplasm (on the left). (original magnification; 12000×).

In order to morphologically establish the presence of the virus in MCPyV-DNA positive samples electron microscopy was performed. Glutaraldehyde is the preferred fixative for electron microscopy. Unfortunately, there was only formalin fixed and paraffin embedded or frozen tissue (without cryoprotection) of MCC present in our archive. These conventional fixation and embedding methods are known to cause various ultrastructural artefacts depending upon several parameters [19]. Due to these artefacts suboptimal morphology was obtained and only one of the MCC samples could be used for ultrastructural investigation (patient no. 4). Electron microscopy of this sample revealed both intranuclear and intracytoplasmic viral particles. The viral particles were stained by electrondense reaction products and measured approximately 50 nm, being compatible with viral polyoma particles. Nuclear localization of loose viral particles, penetration through the nuclear membrane and intracytoplasmic accumulation of capsids was visualized (Figure 1).

The SCLC patients had a mean age of 59 yrs, and 50% were male. The ten SCLC samples were tested for MCPyV-DNA using the LT3 primer set and all tested negative.

The control patients had a mean age of 56 and 50% were male. Of the eight control tissue samples from different patients, none tested positive by MCPyV-DNA PCR.

## Discussion

In this study we provide evidence that only small cell neuroendocrine carcinomas from the skin and not from the lung contain MCPyV-DNA. This suggests that MCPyV has a restricted tropism. MCPyV-DNA was found in the primary and the metastatic tumour of one patient suggesting viral DNA integration. The ultrastructural identification of MCVyP viral particles in primary MCC tumour cell cytoplasm and nuclei in another patient, suggests active viral replication.

In our small patient group we found 40% of the MCC patients positive for MCPyV-DNA, which is more or less similar to data reported in previous studies (43–85%) [6], [11]–[13]. As the viral DNA seems to be able to integrate into the host genome, as was evidenced by Feng et al. and suggested by us, based on our finding of the MCPyV positive metastasis in one patient, a causal relationship between MCPyV infection and the development of MCC is realistic. However until now, on average, only half of the MCC patient populations studied are MCPyV-DNA positive. MCPyV is a member of the polyomavirus group, belonging to the family of the Papovaviridae. Another member of this family are the papillomaviruses and some intriguing similarities between the human papillomaviruses (HPV) and MCPyV may explain the relatively low prevalence of MCPyV in MCC.

First of all the causal relationship between HPV and the development of cervical cancer has been proven [20] and the integration of the HPV-DNA into the host genome is the main feature of oncogenesis. In parallel to our demonstration of MCPyV-DNA in MCC metastases, HPV-DNA has been detected in the metastases of HPV positive invasive cervical carcinomas as well [21]. Furthermore, about 60% of the cervical cancers are positive for HPV 16, the remaining cancers are caused by a broad variety of at least 12 other HPV genotypes [22]. In accordance with cervical carcinoma, the MCCs that tested negative for MCPyV may be caused by other, yet unidentified, MCPyV genotypes, not detected by the specific primer set used in studies so far. This might also explain the much higher proportion of MCPyV positive MCC tumours found in North American patients (43%) compared to Australian patients (24%) [12].

On the other hand this difference could also be due to higher sun exposure in the Australian population. Sun-exposure is an etiological factor in MCC, as evidenced by the finding of UV-related mutations in for instance TP53 in MCC, and clinical association of MCC with other non-melanoma skin cancers [23], [24].

The 40 to 85% range in MCPyV positive MCC may also be explained by the existence of two different but parallel oncogenic pathways, one MCPyV related and one MCPyV non-related pathway. A similar theory of two separate pathways leading to vulvar carcinoma, involving the polyomaviruses-related human papillomaviruses has been suggested.

First, a human papillomavirus (HPV)-dependent pathway, in which premalignant stages of vulvar cancer are the classic vulvar intraepithelial neoplasia (VIN) lesions. Second, an HPV-independent pathway, associated with differentiated VIN III lesions and/or lichen sclerosus [25].

In SCLC, although morphologically almost indistinguishable from MCC, both consisting of small cells with neuroendocrine features and both showing an aggressive behaviour, no MCPyV-DNA could be detected. However, although unlikely, the possibility of unknown strains of MCPyV related to SCLC cannot be ruled out completely.

We are the first to demonstrate the actual presence of the MCPyV within the MCC tumour cell nuclei and cytoplasm morphologically by using electron microscopy. The finding of multiple particles, in the cytoplasm as well as in the nuclei, suggests active viral replication. It demonstrates MCPyV viral particles to be present in MCC tumour cells. Unfortunately, due to fixation of the processed materials, electron microscopy could only be performed on one primary MCC in our study.

In conclusion, we found MCPyV-DNA in 40% of the MCCs and in the metastasis of one patient. MCPyV was found both in an integrated form and, as viral particles as evidenced by electron microscopy in another patient. In the morphological similar SCLC, MCPyV-DNA could not be detected which either suggests a different oncogenic pathway or the existence of yet unidentified MCPyV genotypes. When transforming and immortalizing properties of MCPyV are demonstrated, this will ultimately prove its carcinogenic nature in humans.
